# 5′ isomiR variation is of functional and evolutionary importance

**DOI:** 10.1093/nar/gku656

**Published:** 2014-07-23

**Authors:** Geok Chin Tan, Elcie Chan, Attila Molnar, Rupa Sarkar, Diana Alexieva, Ihsan Mad Isa, Sophie Robinson, Shuchen Zhang, Peter Ellis, Cordelia F. Langford, Pascale V. Guillot, Anil Chandrashekran, Nick M. Fisk, Leandro Castellano, Gunter Meister, Robert M. Winston, Wei Cui, David Baulcombe, Nick J. Dibb

**Affiliations:** 1 Institute of Reproductive and Developmental Biology, Department of Surgery and Cancer, Imperial College London, Du Cane Road, London W12 ONN, UK; 2 National University of Malaysia, Kuala Lumpur, Malaysia; 3 Department of Plant Sciences, University of Cambridge, Cambridge CB2 3EA, UK; 4 Wellcome Trust Genome Campus, Hinxton, CB10 S1A, UK; 5 University of Queensland Centre for Clinical Research, Brisbane, Australia; 6 Imperial Centre for Translational and Experimental Medicine (ICTEM), Department of Surgery and Cancer, Imperial College London, Du Cane Road, London W12 ONN, UK; 7 Max-Planck-Institut für Biochemi, Am Klopferspitz 1, D-82152 Martinsried, Germany; 8 University of Regensburg, Universitaetsstrasse, Germany

## Abstract

We have sequenced miRNA libraries from human embryonic, neural and foetal mesenchymal stem cells. We report that the majority of miRNA genes encode mature isomers that vary in size by one or more bases at the 3′ and/or 5′ end of the miRNA. Northern blotting for individual miRNAs showed that the proportions of isomiRs expressed by a single miRNA gene often differ between cell and tissue types. IsomiRs were readily co-immunoprecipitated with Argonaute proteins *in vivo* and were active in luciferase assays, indicating that they are functional. Bioinformatics analysis predicts substantial differences in targeting between miRNAs with minor 5′ differences and in support of this we report that a 5′ isomiR-9–1 gained the ability to inhibit the expression of DNMT3B and NCAM2 but lost the ability to inhibit CDH1 *in vitro*. This result was confirmed by the use of isomiR-specific sponges. Our analysis of the miRGator database indicates that a small percentage of human miRNA genes express isomiRs as the dominant transcript in certain cell types and analysis of miRBase shows that 5′ isomiRs have replaced canonical miRNAs many times during evolution. This strongly indicates that isomiRs are of functional importance and have contributed to the evolution of miRNA genes.

## INTRODUCTION

Gene expression is subject to extensive regulation by small RNAs, which were first discovered in plants and invertebrates and have key roles in epigenetic regulation, gene transcription and RNA silencing (1,2). The human genome contains over 2000 genes that encode miRNAs of 19 to 26 bases in length that regulate most developmental and physiological processes, including some pathological processes (3). Mature miRNAs are processed from primary transcripts in a two-step process involving the RNase III endonucleases Drosha in the nucleus and Dicer in the cytoplasm, resulting in a miRNA duplex consisting of the miRNA and a complementary star miRNA (4,5). The miRNA or occasionally the star miRNA is then bound within the RISC complex (RNA-induced silencing complex) to one of four Argonaute proteins, in such a way that all of the miRNA sequence with the exception of the very end bases, may anneal to target sequences (6).

MicroRNAs anneal to mRNAs that have complementary target sequences in order to effect RNA silencing, either through the inhibition of RNA translation or by the induction of mRNA degradation (7,8). However, examples of miRNAs that enhance the translation of certain proteins have also been found (9,10). Full complementarity between the miRNA and its target sequence is not required for the inhibition of translation although perfect complementarity is often found to a seed sequence region from bases 2 to 7 of a substantial number of miRNAs (11,12).

A number of experimental analyses indicate that several hundred mRNAs can be suppressed by a single miRNA. The identification of key mRNA targets of individual miRNAs is challenging and a number of genetic and biochemical approaches are being used to address this problem (13–15). Programs such as targetscan (http://www.targetscan.org) and targetscan custom (http://www.targetscan.org/vert_50/seedmatch.html) that predict mRNA targets of miRNAs are very helpful in this regard and are based upon nucleic acid complementarity and other factors such as evolutionary conservation of predicted mRNA target sites. Predictive programs currently use single miRNA sequences listed in miRBase; however, many miRNAs are generated as a family of related isomers that differ by a small number of bases at the 5′ and 3′ end of the miRNA, termed 5′ or 3′ isomiRs (16,17). IsomiRs are most probably generated by variation in processing by Drosha and/or Dicer enzymes, although other processing enzymes may also be involved (16). 5′isomiRs can in theory target different transcripts compared to the canonical miRNA due to shifts in the critical 5′ seed region from the second to seventh nucleotide of the miRNA (18) and so the available software may underestimate the impact of miRNA isoforms upon target gene regulation.

There is good reason to believe that isomiRs are active *in vivo* because they co-immunoprecipitate with Ago proteins and are also active in luciferase and cleavage assays *in vitro* (2,17,19–20). It is less clear how much isomiRs contribute to the functional repertoire of a miRNA gene (16). There is some evidence that isomiRs confer additional mRNA targeting (21–23). Conversely, it has been cogently argued that isomiRs and canonical miRNAs may generally target common mRNAs and that this is beneficial because of the expected reduction in off-target effects (20). It has proven difficult to determine the importance of isomiRs both because of the limited tools that are available for modulating the levels of specific isomiRs (16) and because of the overlapping and redundant nature of most miRNA genes (24).

Here, we sequenced small RNAs from human embryonic stem cells (hESCs), neural stem cells (NSCs) and human mesenchymal stem cells (hMSCs). Analysis of the sequencing data shows that many miRNAs include isomiRs, which were found to co-immunoprecipitate with endogenous Argonaute protein and to be active *in vitro*, indicating that they are functional. We also found that the isomiR to canononical miRNA proportion often differed between cell and tissue types. Bioinformatic analysis predicts that 5′ isomiRs can target large numbers of mRNAs in addition to the ones targeted by the canonical miRNA. *In vitro* luciferase assays validates these predictions and we report that an isomiR-9–1 has gained the ability to inhibit the expression of DNMT3B and NCAM2 but has lost the ability to inhibit CDH1. We were able to use these differences in targeting to construct sponges that were specific for miR-9 or its 5′isomiR. We discuss that 5′isomiRs have frequently replaced miRNAs during evolution, which is supportive of their functional and evolutionary importance.

## MATERIALS AND METHODS

### MicroRNA library cloning

#### 454 sequencing for hMSCs miRNA library and Solexa sequencing for hESCs and NSCs miRNA libraries

A total of 500 μg of total RNA was extracted from first trimester human foetal MSC at passages 5–6 using Trizol (Invitrogen) and ∼10 μg of total RNA of hESCs and NSC were enriched for small RNAs using the miRVana kit (Ambion) according to the manufacturer's instructions. Polymerase chain reaction (PCR) amplification and deep sequencing was performed as specified by (25). Briefly, small RNAs were excised from a 15% 7M Urea polyacrylamide gel electrophoresis (PAGE) and extracted with 0.3 M NaCl. RNAs were ligated to 5′ and 3′ adapters for PCR amplification using 454-forward (5′-GCC TCC CTC GCG CCA TCA GCA GCC ATG GGA ATT CCT CAC TAA-3′) and 454-reverse (5′-GCC TTG CCA GCC CGC TCA GAC AGT CCA TGG ATT G-3′) primers. PCR products were separated on a 10% native PAGE and the amplified ligation products were excised and sequenced with 454 or Solexa technologies. The H1 and H1 neural microRNA libraries are deposited at NCBI SRA submision numbers SRX547311 and SRX548700 and the MSC data is deposited at NCBI GEO GSE58734.

### Neural differentiation

Neural differentiation from hESC line H1 (WiCell) was performed as described (26). Briefly, hESCs were split by ethylenediaminetetraacetic acid (EDTA) (Ambion) and cultured in N2B27 (1:1 mix of D-MEM/F12 supplemented with N2 and Neurobasal medium supplemented with B27, all from Gibco) with 100 ng/ml mouse recombinant noggin (R&D Systems), on a matrigel (BD Biosciences) or poly-L-lysine/Laminin (Sigma-Aldrich) coated plate. Subsequently, cells were split using collagenase and cultured in N2B27 and noggin. After about 3–4 weeks, cells were split using TrypLE^TM^ (Gibco) and cultured in N2B27, supplemented with 20 ng/ml basic fibroblast growth factor (bFGF) (PeproTech).

### Northern blotting

Total RNA (30–50 μg) was separated on a 15% 7M Urea PAGE and transferred to Hybond N+ (Amersham Biosciences) membranes by semi-dry transfer for 30 min at 3.5 mA per cm^2^. Membranes were cross-linked at 1200 Joules/m^2^ and stored in the dark. MiRNA complementary probes were end-labelled with gamma-P^32^-adenosine triphosphate using T4 polynucleotide kinase (New England BioLabs). The membranes were hybridized at 42°C with 7 ml of hybridization buffer (Denhardt's, 0.1% sodium dodecyl sulphate (SDS), 2 × Saline sodium citrate (SSC)) for a minimum of 2 h or overnight. Membranes were washed twice at room temperature with 2 × SSC and 0.1% SDS for 5 min and exposed on x-ray film with an intensifying screen at −80°C for a minimum of 48 h. Digoxigenin labelled locked nucleic acid probe (Exiqon) specific to miR-9, miR-302a and let-7d were used in some of the hybridization experiments.

### Ago immunoprecipitation

Cells were lysed in 10 ml of lysis buffer (25 mM Tris-HCl, 150 mM KCl, 0.5% NP-40, 2 mM EDTA, 1 mM NaF, 0.5 mM DTT and 0.01% protease inhibitors) and centrifuged at 10 000 x *g* for 10 min. The supernatant was incubated with 2 mls of Ago antibody hybridoma (13) rotating at 4°C overnight. Subsequently 80 microlitres of sepharose G beads were added to the supernatant for 1 h at 4°C and the beads were washed three times in wash buffer (300 mM KCl, 50 mM Tris-HCl, 1 mM MgCl_2_ and 0.1% NP-40) and once in phosphate buffered saline. RNA was extracted from the beads with 1× volume of phenol and precipitated.

### Construction of pGL3 luciferase—3′UTR constructs

The 3′UTRs of BTG1, CDH1, DNMT3B, Lefty1, PTEN and Rock1 were amplified from human genomic DNA, cloned into a pGEMT-easy vector and sequence verified. pGEMT-easy vector containing the cloned 3′UTR was excised and ligated into XbaI and FseI sites at positions 1934 and 1953, respectively, of pGL3-Control vector (Promega, E1741) to generate luciferase constructs with 3′UTR containing specific microRNA seed target site. NCAM2 and HMGA2 were ligated into pMIR-REPORT vector (Invitrogen) between SpeI and SacI sites at positions 525 and 519. Primer sequences that were used to generate the UTRs by PCR are listed in Supplementary Table S4, as are the predicted target sites and mutations that were made of some of these sites.

### Transfection and luciferase assay

A day prior to transfection, the HEK 293 cells were split into single cells using trypsin-EDTA (Sigma-Aldrich) and seeded at a density of 50 000 cells in a 24-well plate containing DMEM supplemented with 10% FCS, 1% penicillin/streptomycin (Sigma-Aldrich) and 1% Glutamine (Sigma-Aldrich). Transfection was performed the next day, following the manufacturer's protocol. In brief, 200–400 ng of reporter vector (pGL3, Promega; pMIR-report, Invitrogen) and increasing concentration of miRNA mimic miScipt from 1 to 20 nM (Qiagen) was added to 50 μl of Opti-MEM, as well as 2 μl of HiPerfect (Qiagen).This mixture was incubated at room temperature for 20 min and then added dropwise with gently mixing to the cells in 0.5 ml of freshly replaced media. The cells were incubated at 37°C with 5% CO_2_. All experiments were performed in triplicates, and luciferase expression was measured at 48 h and standardized to Renilla expression.

### Microarray analysis

Overall messenger RNA expression and Ago2 associated RNA was generated by microarray analysis using the HumanWG-6 V3 beadchip (Illumina Inc). The readings obtained from the beadchip platform ranged from a signal of 61.70 to 63796.70; 67.10 to 65461 and 63.0 to 64372.7 (arbitrary units) for hMSC, ESC and NSC, respectively. The microarray data has been deposited here: http://www.ebi.ac.uk/arrayexpress/experiments/E-TABM-1001/

### Western blotting

Protein lysates were prepared from hESC and NSC cells and 20 micrograms loaded per lane. Antibodies: Sox2 (ab97959, Abcam, 1;1000); Oct4 (sc5279, Santa Cruz, 1:500); Pax6(ab2237, Millipore, 1:700), β-Actin (A5441, Sigma, 1:5000).

### Generation of pcDNA3.1(+)-CDH1 and -DNMT3B sponges

Target sites of miR-9 within the 3′UTR of CDH1 and of isomiR-9 within the 3′ UTR of DMN3TB DNA were used to make sponges. DNA fragments consisting of 6 target site repeats of miR-9 or isomiR-9 were synthesized by Eurogentec and were separately ligated into pcDNA3.1(+) under the control of the cytomegalovirus promoter (see Supplementary Table S4 for sequence details).

## RESULTS

### MiRNAs from the same precursor have different 5′ and 3′ lengths

**Figure 1.**
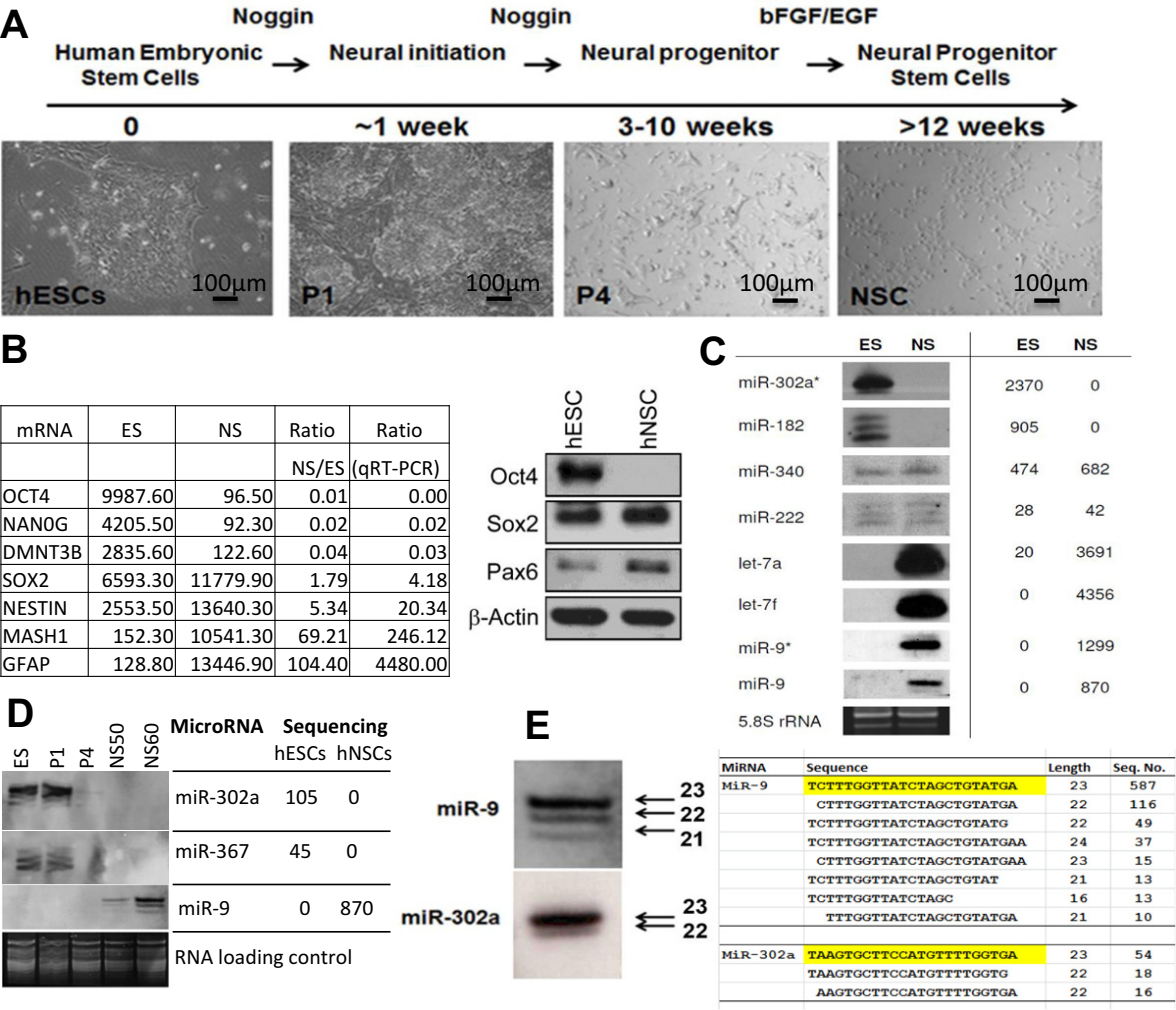
Changes in mRNA and microRNA expression during neural differentiation from hESCs. (A) Morphological features during neural differentiation. (B) Microarray analysis of ES and NS expression of pluripotent and neural markers and confirmation by qRT-PCR and western blotting. (C and D) Northern blotting and sequencing result comparison of the indicated miRNAs during differentiation. (E) Detailed comparison of northern and sequencing results for isomiRs of miR-9 (NSCs) and miR-302a (ESCs). hESCs were differentiated to NSCs according to (26) and cells were collected at four different stages of differention, i.e. hESCs (P0), a week after neural induction (P1), 4 weeks after neural induction (P4) and NSC at passages 40, 50 (NS50) and 60 (NS60). Total RNAs from hESCs (PO) and NSCs (passage 40) were prepared for miRNA sequencing and microarray analysis. These samples and other NSC passages were also analysed by northern blotting, qRT-PCR and western blotting. ESC, embryonic stem cells; NSC, neuronal stem cells.

hESCs are derived from the inner cell mass of a blastocyst and are able to divide indefinitely and to differentiate along the ectoderm, mesoderm and endoderm lineages. We induced hESC *in vitro* differentiation along the neural lineage with Noggin then bFGF and epidermal growth factor (EGF) (Figure 1A) (26). Microarray analysis (Figure 1B, Supplementary Table S1) confirmed that differentiation had occurred and as expected pluripotency markers such as Oct4, Nanog and lin28A were present in hESCs and at the early stages of differentiation, while Nestin, which is expressed mainly by nerve cells, was seen after differentiation. Differentiation was also confirmed by quantitative reverse transcriptase (RT)-PCR and western blotting (Figure 1B, Supplementary Table S1).

**Figure 2.**
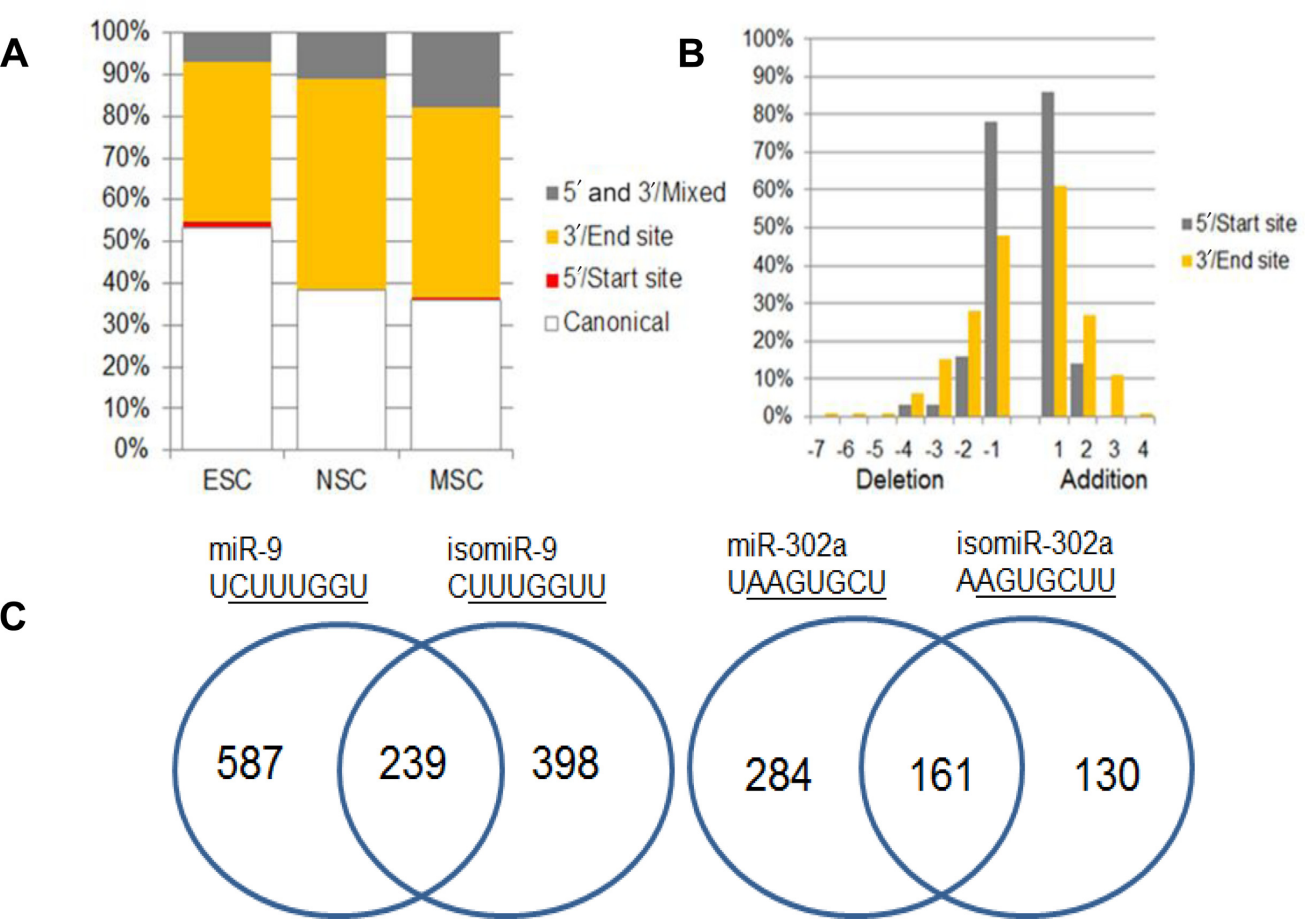
The distribution of 5′ and 3′ isomiRs in hESCs, NSCs and MSCs. (A) A bar graph illustrating the percentage of isomiRs with 5′or 3′ changes compared to the dominant miRNA for ESC, NSC and MSC miRNA libraries. (B) Additions and deletions at the 5′ and 3′ ends for ESC, NSC and MSC miRNAs combined and expressed as a percentage. (C) Venn diagrams comparing the predicted targets of mir-9–1 and the most common 5′ isomiR-9 and similarly for miR-302a. Predictions were made by TargetScanHuman (canonical) and TargetScan custom (isomiRs).

To investigate the role of miRNAs in this process, we cloned and sequenced small RNA libraries from hESCs prior to differentiation and from differentiated neural progenitor stem cells (hNSCs) at passage 40. We also made a miRNA library from hMSCs, see Materials and Methods. Table 1 gives details of the libraries, the complete list of sequenced miRNAs are recorded in Supplementary Table S2. There were striking differences in expression levels of many miRNAs between hESC and derived NSCs, as well as hMSCs, these differences were confirmed by northern blotting (Figure 1C and D). The northern blots also confirmed the observation that most miRNA genes in our libraries produced isomiRs rather than a single miRNA product (Supplementary Table S2). Figure 1E illustrates that the ratios of the isomiR bands for miR-9 and miR-302a that we detected by northern blotting correspond well with the sequencing results. About half of the miRNAs we sequenced had template deletions or additions of bases at the 3′ end compared to the most common miRNA sequence and a smaller but substantial percentage had 5′ alterations (Figure 2A). A greater percentage of 5′ end changes were confined to single base additions or deletions compared to the 3′ end (Figure 2B), indicating that 5′ changes are under greater constraint. Figure 2C illustrates that minor changes at the 5′ ends of miR 9–1 and 302a have substantial impacts upon their predicted targets. Overall, 31.2–46.4% of the predicted targets of the most abundant 5′isomiRs in our three libraries are not predicted targets of their canonical miRNAs and only 22% of the predicted targets are, on average, in common between miRNA and isomiR pairs (Supplementary Table S3).

**Table 1.** Description of the miRNA sequencing libraries

Cell type	Total reads	No. of reads	Unique reads	No. of miRNAs*	
		(15–28 nuc)	(15 – 28 nuc)		
MSC	253 791	25 724	6043	95	454 Seq
ESC	1 697 514	1 276 916	24 534	92	Solexa
NSC	2 345 364	1 274 244	23 426	100	Solexa

*Sequenced more than five times. Unique reads: non-redundant, non-overlapping sequences reads.

### Expression of isomiRs in cell lines and tissues

**Figure 3.**
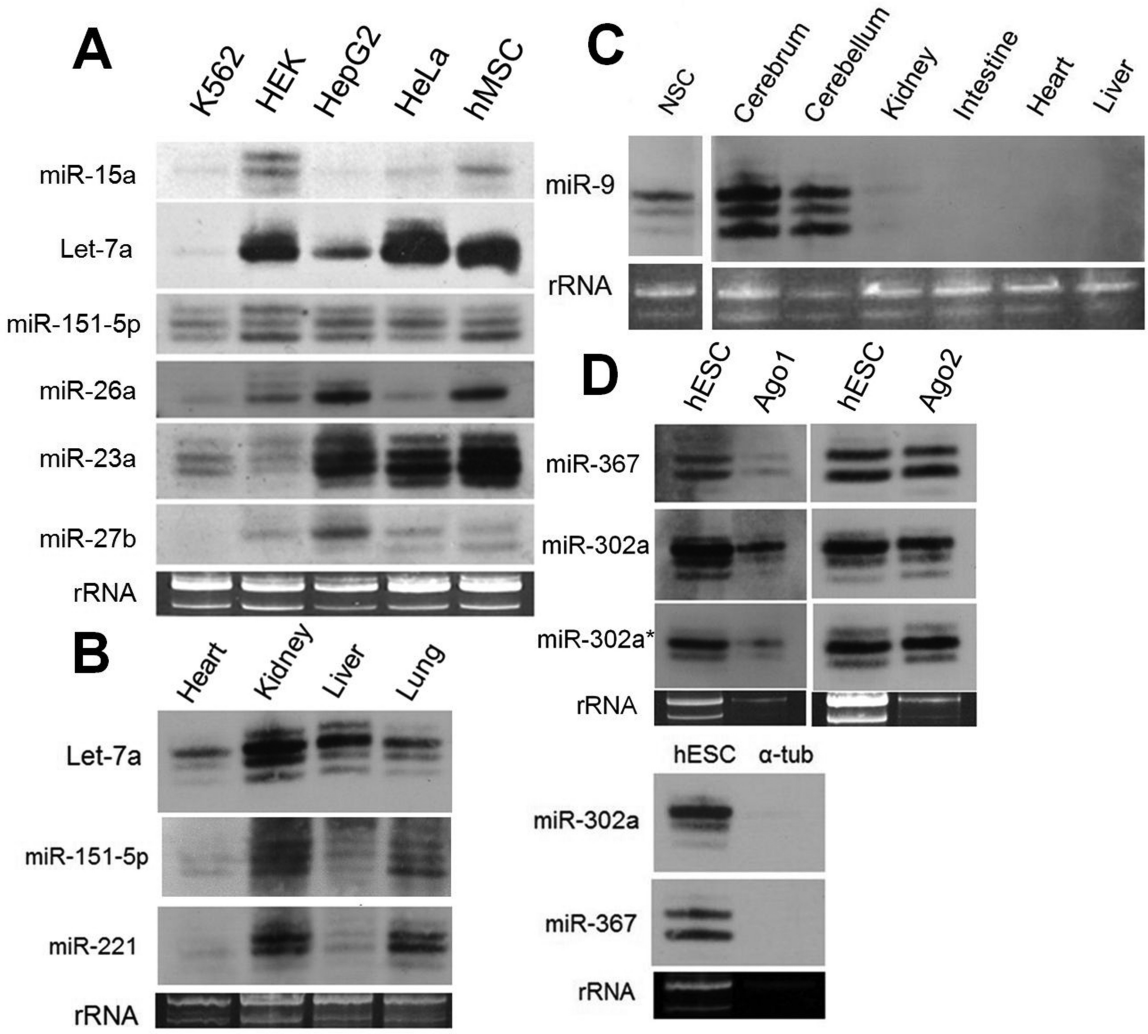
Northern blot analysis of isomiRs in human cell lines and mouse tissues. (A) Human cell lines, (B and C) mouse tissues, (D) human ESC cells before and after immunoprecipitation of Ago1 and Ago2. α-tub, anti-alpha tubulin (negative control). A total of 20 ug of total RNA was loaded per lane. Loading controls were stained with ethidium bromide.

IsomiRs were readily detected in a variety of human cell lines and mouse tissue types by northern blotting, confirming that isomiRs are commonly expressed *in vivo* (Figure 3). The relative ratio of isomiRs encoded by the same miRNA gene varied between cell types, for example, miR-151 and 27b show clear differences across human cell lines and the isomiRs of let-7a and 221 differ across mouse organs (Figure 3A and B).

### IsomiRs associate with Ago1 and Ago2

We tested whether isomiRs were associated with Argonaute (Ago) proteins *in vivo* by comparing northern blotting results for isomiRs present in total RNA samples against isomiRs that were first immunoprecipitated with antibodies against Ago1 or Ago2. The results for all three cell types hESC, hNSC and hMSC (Figure 3D, Supplementary Figure S1), show that isomiRs were co-immunoprecipitated with either Ago1 or Ago2. As expected immunoprecipitation with anti-alpha tubulin did not precipitate miRNAs (Figure 3D) and we further confirmed specificity by showing that a distinctive subset of mRNAs were co-precipitated with anti-Ago from hMSCs (Supplementary Figure S1).

### 5′ and 3′ isomiRs are functional *in vitro*

**Figure 4.**
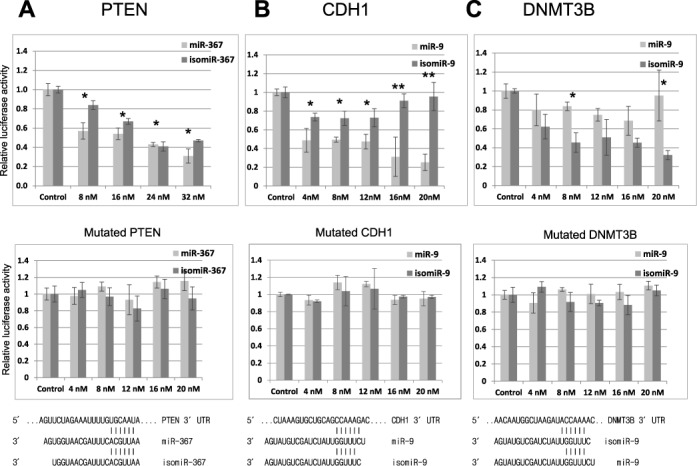
5′ and 3′ isomiR analysis in luciferase assays. (A) Top panel: the 3′UTR of PTEN was cloned into the luciferase vector pGL3 (Promega) and its relative luciferase acitivity is plotted following its transfection (400 ng) into HEK293 cells with the indicated concentrations of miR-367 and a 3′ isomiR. Middle panel: repeat luciferase assay following mutation of the predicted seed target site for miR-367 within the 3′UTR of PTEN. Bottom panel: illustration of the sequences and expected alignment of miR-367 and isomiR-367 against the 3′UTR of PTEN. (B and C) Top panel: identical analyses of cloned regions of the 3′UTR of CDH1, which has a predicted target site for miR-9 and for DNMTB3, which has a predicted target site for a 5′isomiR of miR-9. Middle panels: repeat luciferase assays following mutation of the predicted seed target sites within the 3′UTRs. Bottom panel: illustration of the sequences and expected alignments of miR-9 and 5′isomiR-9 against the 3′UTRs of CDH1 and DNMT3B. The 3′UTRs regions that were chosen and the seed target site mutations are described in Supplementary Table S4. Error bars represent the standard deviation obtained from three independent experiments, * and ** represent statistical significance at the levels of *P* < 0.05 and *P* < 0.0001, respectively. Renilla luciferase was used as internal control to standardize against all firefly luciferase activities. *n* = 3. Note for top panels B and C the statistical difference is between single columns for miR-9 and isomiR-9, whereas for A the statistical difference is between the treatments and the control column pairs.

In order to further test whether isomiRs are functional, we constructed luciferase reporter vectors with the 3′UTR mRNA of potential targets of miR-9, miR-302a, miR367 and their corresponding isomiRs (Supplementary Table S4). We chose these miRNAs because they are among the most abundant miRNAs expressed in hESCs (miR-302 and 367) or NSCs (miR-9–1) and because their isomiRs are co-expressed at levels that are comparable to most of the canonical miRNAs in our libraries (Supplementary Table S2). There are many predicted targets of these miRNAs (targetscan) and their derived isomiRs (targetscan custom) and Table 2 lists the targets that we chose on the basis of their possible biological importance and also summarizes the results of our luciferase assays (Figure 4, and Supplementary Figure S2).

**Table 2.** Summary of the luciferase assay results for the targeting of the indicated 3′UTRs by the indicated miRNAs and isomiRs. The brackets mark targetscan predictions that are discordant with the results.

No	mRNA	MiRNA	Prediction	Luc assay	Notes
1	PTEN	miR-367	√	√	New target
		3′ isomiR-367	√	√	
2	BTG2	miR-367	(√)	X	
		3′isomiR-367	(√)	X	
3	CDH1	miR-9	√	√	Confirmation (ref)
		5′ isomiR-9	X	X	(Ma et al., 2010)
4	DNMT3B	miR-9	X	X	New target
		5′ isomiR-9	√	√	
5	NCAM2	miR-9	X	X	New target
		5′ isomiR-9	√	√	
6	HMGA2	miR-9	√	√	New target
		5′ isomiR-9	(X)	√	
7	BTG1	miR-302a	√	√	New target
		5′ isomiR-302a	(X)	√	
8	Rock1	miR-302a	X	nt	New target
		5′ isomiR-302a	(√)	X	

The 3′UTR of PTEN is a predicted target of both hsa-miR-367 and a common 3′ isomiR of 367 that we sequenced in hESCs (Supplementary Table S2). Our titration shows that the 3′ isomiR of hsa-miR-367 was an equally effective inhibitor of PTEN as miR-367 (Figure 4A). We confirmed that mutation of the predicted target site within the 3′UTR of PTEN prevented repression by both miR-367 and the 3′ isomiR (Figure 4A, middle and bottom panels). By contrast we were unable to confirm the prediction that BTG2 is a target of miR-367 (Table 2, Supplementary Figure S2).

### 5′isomiRs can target different mRNAs

Figure 4B and C analyses two genes CDH1, which is a known target of miR-9 (27) but is not a predicted target of isomiR-9 and DNMT3B, which is a predicted target of isomiR-9 but not of mir-9 (Table 2). We chose these two genes because they are expressed in hESCs and are downregulated upon differentiation, which also corresponds with the appearance of miR-9 and isomiR-9 (Figure 1B and D). Luciferase assays confirmed that the 3′UTR of CDH1 is a target of miR-9, but our titration clearly shows that isomiR-9 was not able to repress luciferase activity as efficiently (Figure 4B). By contrast, a similar titration experiment shows that the 3′UTR of DNMT3B was a target of isomiR-9 but not miR-9 (Figure 4C). Our titrations indicate that isomiR-9 is an equally good inhibitor of DNMT3B as miR-9 is of CDH1. As a control we mutated two small regions within the 3′UTRs of CDH1 and DNMT3B that are the predicted binding sites for the seed regions of miR-9 and isomiR-9, respectively, this markedly reduced luciferase inhibition in both cases (Figure 4B and C, middle and bottom panels).

### MiRNA sponges for isomiRs

**Figure 5.**
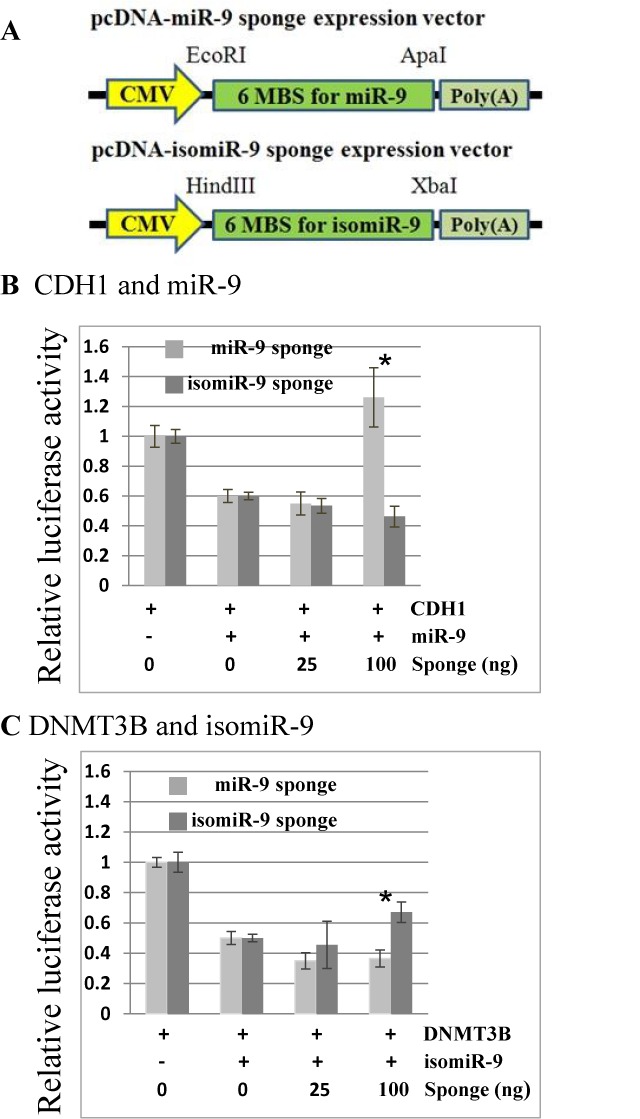
Sponge inhibitors of miR-9 and isomiR-9. (A) Outline of sponge constructs pcDNA-miR-9 amd pcDNA-isomiR-9 (see Materials and Methods). HEK293 cells were transfected with the indicated concentrations of each sponge vector with either (B) pGL3-CDH1–3′UTR (400 ng) and miR-9 (12 nM) or (C) pGL3-DNMT3B-3′UTR (400 ng) and isomiR-9 (12 nM). All results were normalized by renilla luciferase. For sponge sequences see Supplementary Table S4. Error bars represent the standard deviation obtained from three independent experiments, * represents statistical significance (between sponges) at *P* < 0.05.

In order to strengthen these results, we constructed two expression vectors that contain six repeated binding sites for either miR-9 or isomiR-9. These binding sites have the same sequence as the target sites within the 3′UTRs of CDH1 or DNMT3B (Figure 5A, Supplementary Table S4). The first two column pairs of Figure 5B doubly repeat the observation that 12 nM miR-9 can inhibit the expression of luciferase mRNA when it is fused to the 3′UTR of CDH1. The next two column pairs show that inhibition by 12 nM miR-9 can be relieved by sufficient amounts of a miR-9 sponge (100 ng) but not by an isomiR-9 sponge (Figure 5B). By contrast, Figure 5C shows that the isomiR-9 but not the miR-9 sponge can relieve the inhibition of the DNMT3B luciferase expression vector by isomiR-9. Similarly, we identified NCAM2 as a target of isomiR-9 but not miR-9 and also showed that repression by isomiR-9 could be rescued by an isomiR-9 sponge (Supplementary Figure S2).

Overall, we confirmed predicted differences in targets between miRNA and 5′ isomiR pairs in three out of six cases by luciferase assays (Table 2, rows 3, 4 and 5 versus rows 6, 7 and 8). There were two false negative and three false positive predictions, which are enclosed in brackets (Table 2). In addition, the 3′UTRs of BTG1 and HMGA2 were confirmed as predicted targets of both miRNAs and 5′isomiRs of miR-302a and miR-9, respectively.

### 5′ isomiR selection during evolution

**Figure 6.**
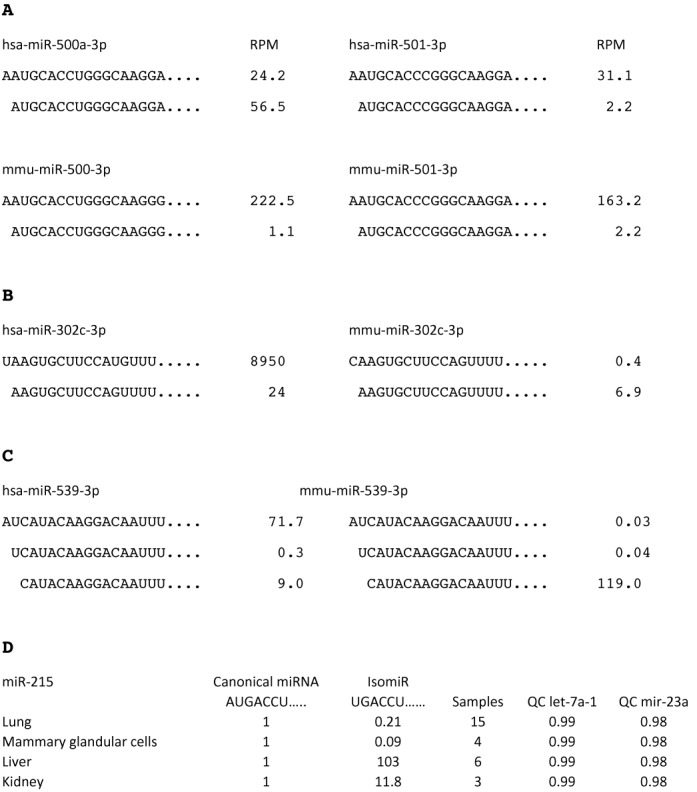
IsomiR switching. (A) An example of 5′isomiR switching for hsa-mir-500a-3p. An isomiR of hsa-mir-501 (which is a paralogue of hsa-miR-500) and the mouse gene orthologues mmu-mir-500 and 501 (AUGCACCC…..) is expressed as a canonical miRNA by hsa-mir-500a. Only the most highly sequenced isomiR and miRNA (lead strands) are shown for these genes, there are less common isomiRs for these genes that are not shown. RPM is sequencing reads per million in total. The mir-500 family of miRNA genes are found only in mammals and in addition to *Homo sapiens*, the miR-500 genes of *Gorilla gorilla, Sus scrofi* and *Canis familiaris* also express AUGCACCU…. as the canonical miRNA. The remaining mammals for which there is sequencing data (*Mus musculus* and *Rattus norvegicus*) express AAUGCACCA …. as the canonical miRNA and AUGCACCA as an isomiR. For all other members of the human miR-500 family (miR-500b, 501 and 502) AUGCACCU is expressed as an isomiR and AAUGCACCA … as the canonical miRNA. (B) 5′isomiR to miRNA switch for mmu-mir-302c-3p. The miRNA AAGUGCUU….. is expressed as a canonical miRNA by mmu-miR-302c but as an isomiR by hsa-miR-302c and all of the remaining members of the miR-302 family of man and mouse. (C) An isomiR of hsa-mir-539 (CAUACAA….) is expressed as a canonical miRNA by mmu-mir-539 and by contrast an isomiR of mmu-mir-539 (AUCAUACAA….) is expressed as the canonical miRNA by hsa-mir-539. All data are taken from miRBase release 19, (30) and confirmed in miRGator v.3.0 for human sequences. In particular, miRGator confirmed that the dominant transcript of hsa-mir-500a has a 5′ sequence AUGCACCC….. in most cell types (29). (D) Table showing that hsa-mir-215 encodes an isomiR (UGACCU) that is the dominant transcript in the liver and kidney. The miR-215 canoncial sequence (miRBase, (30)) is assigned a value of 1 for each tissue type and the isomiR value equals the total number of isomiR sequence reads/total number of canonical sequencing reads for each tissue. Data compiled from 15, 4, 6 and 3 sequencing reads from lung, mammary glandular cells, liver and kidney currently deposited in miRGator v3.0 (29). The last two columns QC let-7a-1 and QC miR-23a are quality controls of the sequencing data. We analysed the invariant miRNAs let-7a-1 by dividing the total number of sequencing reads for the canonical miRNA by the total number of sequencing reads. We similarly analysed has-miR-23a. This method can identify samples that suffer from incomplete sequencing reads, see Supplementary Table S5 for further details.

We next asked whether 5′isomiRs have been selected during evolution, as this would also indicate that isomiRs are of functional importance. Figure 6A shows an example of two paralogous human miRNA genes, hsa-mir-500a and 501, together with the equivalent orthologous mouse miRNA genes mmu-mir-500a and 501 (data from miRBase). It can be seen that the most common isomiR of hsa-mir-501, and the two mouse miRNA genes (AUGCAC…….) is a canonical miRNA for hsa-mir-500a. This indicates that a canonical miRNA may evolve as a result of increases in isomiR frequency. The observation of 5′isomiR switching between hsa-mir-500a and 501 (Figure 6A) is not simply a result of inconsistent sequencing between different samples because this result was extensively confirmed for single samples from a wide range of tissues listed in miRGator (Supplementary Table S5). We found this one example of 5′isomiR to miRNA switching between paralogous human miRNA genes out of 140 gene cluster examples (28) that we screened in both miRBase and miRGator (29,30).

In addition to hsa-mir-500a and mmu-mir-500, we found two other examples of 5′isomiR to miRNA switching between 213 comparisons of human and mouse orthologous miRNA gene families (Figure 6B and C; data from miRBase). These isomiR to miRNA switches are likely to be equivalent to previous reports of seed shifts between miRNA genes from a variety of species (31,32) (Supplementary Figure S3; see Discussion).

We also asked to what extent the same miRNA gene switches 5′isomiR expression between tissues. Figure 6D gives an example of miR-215, in which a 5′isomiR (UGACCU) is expressed as the dominant transcript by far in liver and kidneys. We found four other convincing examples of 5′ isomiR switching (miR-101, 106a, 140 and 4454) between human cell types out of a screen of 295 of the most highly expressed miRNAs in miRGator (29) (Supplementary Table S5).

## DISCUSSION

Over half of the miRNAs genes from our three stem cell libraries were expressed as isomers (isomiRs) that have 5′ or 3′ template differences compared to the dominant canonical sequence (Figure 2A, Supplementary Table S2). The variation we detected is unlikely to be a cloning or sequencing artefact because we observed similar variation in all cases that were tested by northern blotting (Figures 1 and 3). Previous miRNA sequencing projects report the presence of isomiRs and similarly to our experiments demonstrated their association with Ago proteins (2,17,20) (Figure 3). A number of groups report that 3′ isomiR expression patterns differ between cell lines or tissue types and in some cases the changes are as much as 10-fold (33,34). Similarly, we observed differences in isomiR expression between cell types by northern blotting and by analysis of isomiR sequencing databases (Figures 3 and 6).

The 5′ isomiR variants we sequenced occurred at a frequency of only 5–15%, which is lower than the observed 3′isomiR variation of 40–50% between the three libraries (Figure 2). However, 5′ isomiR variation is predicted to have a major impact upon mRNA targeting, with an average of only 22% of predicted targets in common between 5′isomiR and miRNA pairs encoded by the same gene (Supplementary Table S3), which is reflective of the predictive weighting given to the 5′ seed region of miRNAs for target recognition (18). We used luciferase assays to confirm predicted differences between miRNA and isomiR pairs in three out of six cases (Table 2). Our results therefore support the prediction that single nucleotide changes at the 5′end of a miRNA have a substantial and easily measurable impact upon mRNA targeting *in vitro*. We strengthened these results by showing that it was possible to suppress the inhibitory effect of individual isomiRs *in vitro* through the use of isomiR-specific sponge vectors (Figure 5). It is important to note that many of the predicted mRNA targets of isomiRs are unique. For example, isomiR-9 has 398 novel predicted targets compared to miR-9 (Figure 2) and of these 18 are not predicted targets of any other human miRNA. Consequently, isomiR production increases the range of potential mRNA targets.

Our results extend a previous report that miR-133a and a commonly sequenced 5′isomiR preferentially inhibit mRNAs encoded by *Ctgf* and *PgamI*, respectively, in luciferase assays (23). Furthermore, it has also been noted that knock-out of miR-223 in mouse neutrophils causes the depression of some mRNAs that are not predicted targets of miR-223 but instead are predicted targets of a minor 5′isomiR that is also expressed in this cell type (21). Overall, the available evidence strongly indicates that 5′isomiRs are fully functional *in vivo*. Our finding that some 5′isomiRs have become canonical miRNAs during evolution or are the dominant miRNA in certain tissues adds support to the more limited evidence that isomiRs are also important (16).

Gene duplication or *de novo* hairpin formation is considered to be key to miRNA evolution as it provides an opportunity for new miRNA variants to evolve without destroying the tried and tested old variant (28,35). It has also been suggested that new miRNAs are likely to be expressed at low levels initially in order to avoid deleterious targeting effects and to allow the miRNA and selectively advantageous mRNA targets to co-evolve (36). These criteria are both met by initial low level production of isomiRs by single miRNA genes. Figure 6 illustrates how minor isomiRs may become canonical miRNAs during evolution and how gene duplication allows the retention of both old and new miRNA variants. Presumably a lot of the low level 5′isomiR variation that is observed is not of physiological importance; however, the observation that isomiRs may replace miRNAs during evolution illustrates that some of the low level isomiR variation may acquire functionality in the future, as previously argued for small RNA variation in general (36).

Our analysis supports the suggestion (28) that changes in 5′isomiR usage during evolution are responsible for previous observations of seed shifting (31,32). Seed shifting refers to miRNA transcripts that are encoded by orthologous or paralogous genes that have identical seed regions at the DNA level yet produce miRNA transcripts that differ by small templated additions or deletions at the 5′end of a miRNA transcript, presumably due to changes outside the encoded miRNA that affect processing. Wheeler *et al.* (32) identified five such miRNA genes that in a minority of species express dominant miRNAs that have templated 5′ deletions or additions compared to the equivalent miRNA transcripts expressed by most species. Similarly, Marco et al., (31) identified examples of seed shifting by comparing 46 orthologous miRNA genes from *Drosophila melanogaster* and *Tribolium castaneum*. The more extensive sequencing data that is now available shows that all of these novel canonical miRNA transcripts that have seed shifts are also found as 5′isomiRs in many species (Supplementary Figure S3), indicating that seed shifting occurs through the enhanced production of pre-existing 5′isomiR variants.

IsomiR switching would seem analogous to miRNA arm-strand switching, which has been documented to occur between paralogous and orthologous genes and between different tissue types (31,32). It is highly likely that strand switching causes a substantial change in mRNA targeting (31), however, the effect of isomiR switching is less clear. Cloonan *et al.* (20) observe that isomiR and canonical miRNA expression is usually highly correlated and they provide evidence that this is because related isomiRs and miRNAs tend to share similar targets. They also point out that the targeting of a common mRNA by multiple miRNAs might be advantageous because of the expected reduction in off-target effects compared to targeting by a single miRNA species. Similarly, only common targets of miR-101 and a frequently expressed isomiR of miR-101 have been identified, despite the fact that these two miRNAs, show marked differences in expression between cell types (37) (Supplementary Table S5). On the other hand, Fukunaga et al., (22) report that only the longer form of two 5′isomiRs encoded by miR-307 can target the mRNAs for glycerol kinase and taranis in Drosophila ovaries. Anlaysis of the miRGator database indicates that a small percentage of human miRNA genes show substantial differences in their miRNA to 5′isomiR ratios between different cell types (Figure 6D; Supplementary Table S5), this percentage may increase as further tissue specific sequencing data becomes available. Our demonstration that it is possible to construct sponges that are 5′isomiR specific should help to address the question as to whether 5′isomiR switching between tissues purposefully changes the targets of miRNA genes.

## SUPPLEMENTARY DATA

Supplementary Data are available at NAR Online.

SUPPLEMENTARY DATA
