# Supplementary material for: 5′ isomiR variation is of functional and evolutionary importance
Source: Nucleic Acids Res. 2014 Jul 23;42(14):9424–35. doi: 10.1093/nar/gku656 (PMC4132760; doi:10.1093/nar/gku656)
Supplement: SUPPLEMENTARY DATA [file supp_42_14_9424__index.html]

5′ isomiR variation is of functional and evolutionary importance — 5′ isomiR variation is of functional and evolutionary importance — SUPPLEMENTARY DATA 

# 5′ isomiR variation is of functional and evolutionary importance

## SUPPLEMENTARY DATA

**Files in this Data Supplement:**

- SUPPLEMENTARY DATA
- SUPPLEMENTARY DATA
- SUPPLEMENTARY DATA
- SUPPLEMENTARY DATA
- SUPPLEMENTARY DATA
- SUPPLEMENTARY DATA
- SUPPLEMENTARY DATA
- SUPPLEMENTARY DATA
- SUPPLEMENTARY DATA
