# Supplementary material for: 5′ isomiR variation is of functional and evolutionary importance
Source: Nucleic Acids Res. 2014 Jul 23;42(14):9424–35. doi: 10.1093/nar/gku656 (PMC4132760; doi:10.1093/nar/gku656)
Supplement: SUPPLEMENTARY DATA [file supp_gku656_nar-02457-a-2013-File011.doc]

**Supplementary figure and table legends**

**Figure S1**

IsomiRs from hMSC and NSC associate with Argonaute proteins

A) Isomers of let-7a, miR-23a, miR-26a, miR-221 and miR-222 could be detected by northern blotting of total RNA and of RNA immunoprecipitated by anti-Ago1 and anti-Ago2 from hMSCs. B) northern blot of miR-9 before and after Ago1 or Ago2 immunoprecipitation from NSCs. (C,D) Controls showing that a distinct subset of mRNAs are co-immunoprecipitated with Argonaute proteins. We confirmed the specificity of the Ago immunoprecipitations by showing that only a subset of mRNAs from lysates of hMSCs co-precipitated with Ago2 protein. C). The x axis gives the microarray intensity signal for each of the total mRNAs expressed by mesenchymal stem, these are described in detail in Table S1sheet 3 and the y axis gives the microarray signal for the mRNAs that were immunoprecipitated by antibody against Ago2 (Table S1 sheet 4). The majority of mRNAs were not immunoprecipitated but there was considerable enrichment of some 119 mRNA species (Table S1 sheet 4). D) As expected, three representative mRNAs (F2R, ROCK2, BGN) from the list of 119 precipitated mRNAs were also detected in repeat Ago2 immunoprecipitation experiments by RT-PCR, whereas four mRNAs that were not detected in Fig S1C (and see Table S1 sheet 4) were also not detected in repeat experiments - total RNA (+), Ago2 bound RNA (A) and a negative antibody control (-).

**Figure S2**

The 3’UTRs of NCAM2 (A-C) HMGA2 (D,E), BTG1 (F,G), BTG2 (H) and Rock1 (I) were cloned into the luciferase reporter vector pGL3control (Promega) and their relative firefly luciferase activity is plotted following their transfection into HEK293 cells(200 ng) against a titration of the indicated concentrations of miRs and isomiRs. For B) column pairs 2 to 4, HEK293 cells were transfected with 12 nM of isomiR-9 and the indicated amount of miR-9 or isomiR-9 sponge constructs. C,E,G) Control experiments in which the predicted target sites of the 3’UTR of CDH1, DNMT3B and BTG1 expressed by pLG3control were mutated (see Materials and methods). miR-9: UCUUUGGUUAUCUAGCUGUAUGA; isomiR-9: CUUUGGUUAUCUAGCUGUAUGA

All results were normalised by renilla luciferase. Error bars represent the standard deviation obtained from three independent experiments, * and ** represent statistical significance (between miR-9 and iosmiR-9) at the levels of p<0.05 and p<0.0001 respectively.

**Figure S3**

A) Interchange of miR-2 miRNA and an isomiR between paralogous genes of *Bombyx mori*. miR-2 is widespread in invertebrates and (31) identified a small number of miR-2 genes that express a shorter miRNA of sequence UCACAGCCA.... instead of the more common longer miRNA, usually of sequence UAUCACAGCCA.... We report that more extensive sequencing data reveals that the shorter miRNA is also a common isomiR for genes such as bmo-mir-2a-2 (see above), dme-mir-2a; dme-mir-2c and tca-mir-2a (miRBase).

B) Three different isomiRs of miR-10 selected as canonical miRNAs during evolution. miR-10 is a widely expressed miRNA that usually encodes the miRNA UACCCUGUA.... (31) also identified miRNA genes that encode a longer miRNA of sequence UUACCCUGUA.... made by one of two miR-10 genes of both *Haliotis rufescens* and *Lottia gigantean*. We report that orthologous miR-10 genes make a number of isomiRs of which at least three are canonical miRNAs in some species.

C) Likely interchange between isomiRs and miRNAs for paralogous genes cte-mir-745a and 745b during evolution. The miRNA AGCUGCCUGGU.... encoded by cte-mir-745a is expressed as an isomiR by the orthologous miRNA genes mir-22 and mir-980 in other species including *Branchiostom floridae* and *Drosophila melanogaster* (miRBase)

D) Interchange of miR-133 miRNA and isomiR between species. The miR-133 gene is widely expressed either as UUUGGUCCCCU by animal species such as mice or as a shorter 5’ variant UUGGUCCCCU....by most insect species. Some species such as *Branchiostoma floridae*, *Homo sapiens* and *Saccoglossus kowelevskii* make more equal amounts of these variants.

E) Interchange of miR-137 miRNA and isomiRs between species. The miR-137 gene is widely expressed either as UUAUUGCU.... by species such as mouse, human and *C.elegans* whereas the shorter sequence UAUUGCU.... is more prevalent in insects (3,29,30).

F) miR-210 variants in some species are likely to have arisen from isomiR variants. The miR-210 variant CUGUGCGUGU.... is a canonical miRNA in mouse but an isomiR in Cqu. Similarly the variant CUUGUGCGUGU is an isomiR in Ame but the canonical miRNA for species such as *Culex quinquefasciatus* (miRBase) and *Nereis diversicolor* (31).

Ame, *Apis mellifera*; Bfl, *Branchiostoma floridae*; Bmo, *Bombyx mori*; Cqu, *Culex quinquefasciatus*; Cte, *Capitella teleta*; Dme, *Drosophila melanogaster*; Hsa, *Homo sapiens*; Mmu, *Mus musculus*; Ndi, *Nereis diversicolor*; Sko, *Saccoglossus kowelevskii*; Tca, *Tribolium castaneum*.

A. pMIR-NCAM2-3’UTR (200 ng) was co-transfected with miR-9 or isomiR-9 miRNA mimic in HEK 293 cells. IsomiR-9 was able to knockdown the luciferase activity at 12 nM and 20 nM concentration. However, miR-9 was not able to do so as effectively as isomiR-9. All results were normalised by renilla luciferase. B. Rock gene is a false positive predicted target of isomiR-302a. C. BTG1 gene is a false negative predicted target of isomiR-302a.

**Table S1**

**Human embryonic, neural and mesenchymal stem cell mRNA microarray.** The top mRNAs detected by HumanWG-6 V3 beadchip (Illumina Inc) are listed below for two repeats if each cell type (sheets 1 to 3). Sheet 4 - mRNAs isolated from Ago2 immunoprecipitation from human mesenchymal stem cells. An average signal of 150.00 (arbitrary units) from two repeats was taken as the threshold above background and mRNAs signals below this are not included. Sheet 5 - quantitative RT-PCR was used to confirm some of the microarray results presented in Figure 1B (main text). ES – embryonic stem cells, NPC – neuronal progenitor cells at passages 5 or 45.

**Table S2**

MiRNA sequencing results of human embryonic stem cells (hESC), neural stem cells (NSC), human mesenchymal stem cells (hMSC).

**Table S3**

The percentage of common and specific target predictions for miRNA/isomiR pairs expressed by hESCs, NSCs or MSCs. Analysis was performed on some of the miRNAs and the most common isomiR identified by sequencing (Table S2). Predictions by targetscan Columns A to C list the miRNAs and seed regions of the miRNA and most commonly sequenced isomiR (Table S2). The remaining columns show the percentage of common and specific targets for each miRNA/isomiR pair. For example, there are 681 predicted targets for miR-101-3p and its isomiR combined and of these 109 and 158 are specific targets for the miRNA and isomiR respectively and 414 are common targets.

**Table S4**

**Primers and target site mutations**. List of primers that were used to make PCR fragments of the 3’ UTRs of the listed genes prior to cloning into luciferase expression vectors. Also shown are the predicted target sites within the 3’UTRs for the indicated miRNAs and the mutations that were introduced into the predicted target sites for some of the UTRs. **Sponge sequences**. Sequences of six repeated target sites of miR-9 (from the 3’UTR of CDH1) and six target sites of isomiR-9 (from the 3’UTR of DNMT3B) that were used to make the sponges described in Figure 5 (main text).

**Table S5**

Screen of miRGator for possible isomiR switching by a set of 300 human miRNA genes. Sheet 1 - list of miRNA genes that were analysed. These were chosen as they had the highest expression values in miRBase. Sheet 2- columns A to C - a list of 74 tissue types from miRGator that were initially screened for the expression of the miRNAs listed in sheet 1. Sheet 2 columns D to G assess the quality of the sequencing data by dividing the reads for the let-7a 5’ canonical miRNA (UGAGGU….) by the total reads for let-7a. Columns I to K - similar analysis for hsa-mir-23a. Samples 1, 138, 159, 450 and 631 were filtered out by this method and subsequently samples 632-4. Sheet 3 (500a, 501) – this analysis confirms and extends the observation in miRBase of isomiR switching between has-mir-500a and 501 (Figure 6a). Remaining sheets – detailed analysis of individual miRNAs that showed the strongest evidence of isomiR switching between cell types. There is a summary chart for sheets 3 to 7 showing the relative expression levels of the canonical miRNA and most common isomiR across a range of tissues. The let7a and 23a columns are quality checks of the sequencing data similar to those described in sheet 2.
