# Supplementary material for: 5′ isomiR variation is of functional and evolutionary importance
Source: Nucleic Acids Res. 2014 Jul 23;42(14):9424–35. doi: 10.1093/nar/gku656 (PMC4132760; doi:10.1093/nar/gku656)
Supplement: SUPPLEMENTARY DATA [file supp_gku656_nar-02457-a-2013-File015.docx]

**Table S4**

**Primers and target site mutations**

BTG1 UTR

Forward primer ATGCTAGCTGCCATAGTTTGGACAGTAC

Reverse primer ATGGCCGGCCAATGTACAGAGAGCTGGCTG

Size 520bp

miR-302a 3’AGUGGUUUUGUACCUUCGUGAAU5’

isomiR-302a 3’AGUGGUUUUGUACCUUCGUGAA 5’

BTG1 predicted target site ACAAGACUUUUACCUAGCACUUA

BTG1 mutated target site ACAAGACUUUUACCUCGUAUCUG

BTG2 UTR

Forward primer ATGCTAGCTTGGAACCACATGAAAGTCT

Reverse primer ATGGCCGGCCGGTGGCCATCCTGGCCAAAT

Size 698bp

miR-367 3’AGUGGUAACGAUUUCACGUUAA5’

isomiR-367 3’ UGGUAACGAUUUCACGUUAA5’

BTG2 predicted target site AGUUCUCAGUCACUGUGCAAUA

CDH1 UTR

Forward primer ATGCTAGCCTCACTCCTGAATTCAGTTG

Reverse primer ATGGCCGGCCGATCCAAATCAAGATCCTCA

Size 680bp

miR-9 3’AGUAUGUCGAUCUAUUGGUUUCU5’

isomiR-9 3’AGUAUGUCGAUCUAUUGGUUUC 5’

CDH1 predicted target site CUAAAGUGCUGCAGCCAAAGAC

CDH1 mutated target site CUAAAGUGCUGCAGACGUAUGC

DNMT3B UTR

Forward primer ATGCTAGCGCAGAGCCACCTGACTCTTG

Reverse primer ATGGCCGGCCTAATAGGTCCCGTGCAGACT

Size 470bp

miR-9 3’AGUAUGUCGAUCUAUUGGUUUCU5’

isomiR-9 3’AGUAUGUCGAUCUAUUGGUUUC 5’

DNMT3B predicted target site AACAAUGGCUAAGAUACCAAAAC

DNMT3B mutated target site AACAAUGGCCAAGAUGCAACCAC

LEFTY1 UTR

Forward primer ATGCTAGCGTAGCCATCGAGGGACTTGA

Reverse primer ATGGCCGGCCTGGATTGGGGATGCACAA

Size 401bp

miR-302a 3’AGUGGUUUUGUACCUUCGUGAAU5’

isomiR-302a 3’AGUGGUUUUGUACCUUCGUGAA 5’

LEFTY1 predicted target site CUGCACUAUAUUCUAAGCACUUA

PTEN UTR

Forward primer ATGCTAGCGTAGGGTACAAGTTTAATGT

Reverse primer ATGGCCGGCCTAACAAATGGACATCTGATT

Size 417bp

miR-367 3’AGUGGUAACGAUUUCACGUUAA5’

isomiR-367 3’ UGGUAACGAUUUCACGUUAA5’

PTEN predicted target site AGUUCUAGAAAUUUUGUGCAAUA

PTEN mutated target site AGUUCUAGAAAUUUUACGCGUAA

ROCK1 UTR

Forward primer ATGCTAGCGTAGAAGGTTGCACCAACAT

Reverse primer ATGGCCGGCCACATATCCATCAGTGCGGCT

Size 305bp

miR-302a 3’AGUGGUUUUGUACCUUCGUGAAU5’

isomiR-302a 3’AGUGGUUUUGUACCUUCGUGAA 5’

ROCK1 predicted target site GUGAUCAGAUAAGAAGCACUGG

NCAM2 UTR

Forward primer AGTCTAGAACAATATTACAGGGGCTTGA

Reverse primer ATGGGCCCATAGAGCACTTTAGCCACAT

Size 307bp

miR-9 3’AGUAUGUCGAUCUAUUGGUUUCU5’

isomiR-9 3’AGUAUGUCGAUCUAUUGGUUUC 5’

NCAM2 predicted target site AUUGCGUGACCCUAUGACCAAAA

NCAM2 mutated target site AUUGCGUGACCCUAUGGCUUAGG

HMGA2 3'UTR

Forward primer AGTCTAGATAGTCAATCACTGCACTGCA

Reverse primer ATGGGCCCTGGCTCTGTAGGAAGTAGAT

Size 433bp

miR-9 3’AGUAUGUCGAUCUAUUGGUUUCU5’

isomiR-9 3’AGUAUGUCGAUCUAUUGGUUUC 5’

HMGA2 predicted target site UCCUCUGUUUAGAACACCAAAGAU

HMGA2 mutated target site UCCUCUGUUUAGAACUGCACUGCU

**Sponge sequences**

6 target sites in the miR-9 sponge

ACTAGTCGGAAGTGCCTAAAGTGCTGCAGCCAAAGACGATAGTGCCTAAAGTGCTGCAGCCAAAGAACGCGTACGGAAGTGCCTAAAGTGCTGCAGCCAAAGACGATAGTGCCTAAAGTGCTGCAGCCAAAGAACTAGTACGGAAGTGCCTAAAGTGCTGCAGCCAAAGACGATAGTGCCTAAAGTGCTGCAGCCAAAGAAGCTT

6 target sites in the isomiR-9 sponge

ACTAGTCGGAAGAAACAATGGCTAAGATACCAAAACGATAGAAACAATGGCTAAGATACCAAAAACGCGTACGGAAGAAACAATGGCTAAGATACCAAAACGATAGAAACAATGGCTAAGATACCAAAAACTAGTACGGAAGAAACAATGGCTAAGATACCAAAACGATAGAAACAATGGCTAAGATACCAAAAAGCTT
