# Supplementary figures and images for: 5′ isomiR variation is of functional and evolutionary importance
Source: Nucleic Acids Res. 2014 Jul 23;42(14):9424–35. doi: 10.1093/nar/gku656 (PMC4132760; doi:10.1093/nar/gku656)

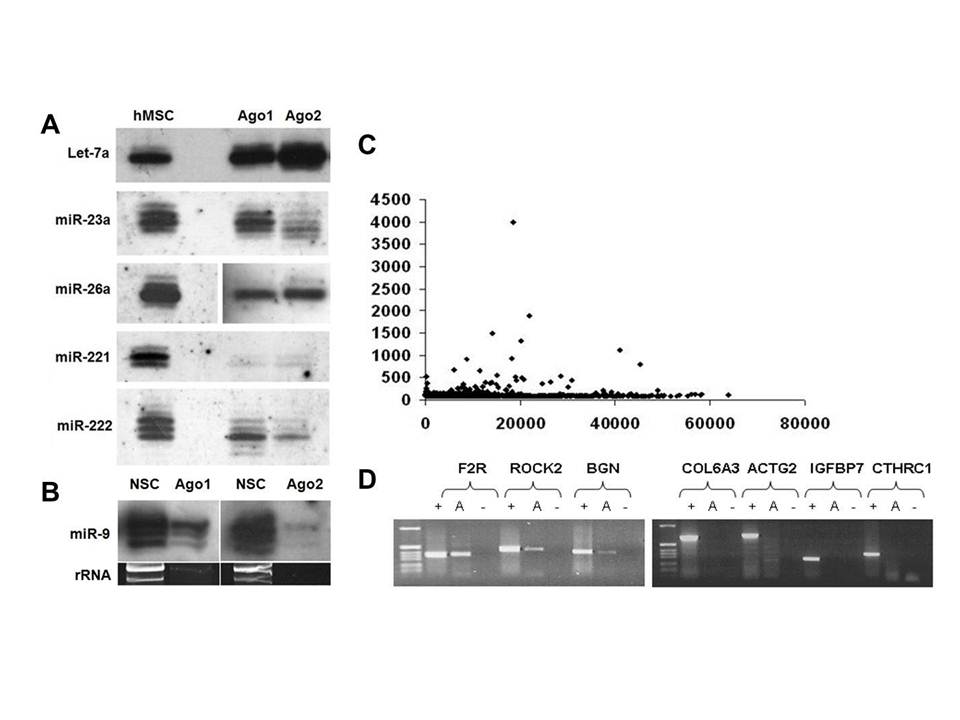

Supplement: SUPPLEMENTARY DATA [file supp_gku656_nar-02457-a-2013-File017.jpg]

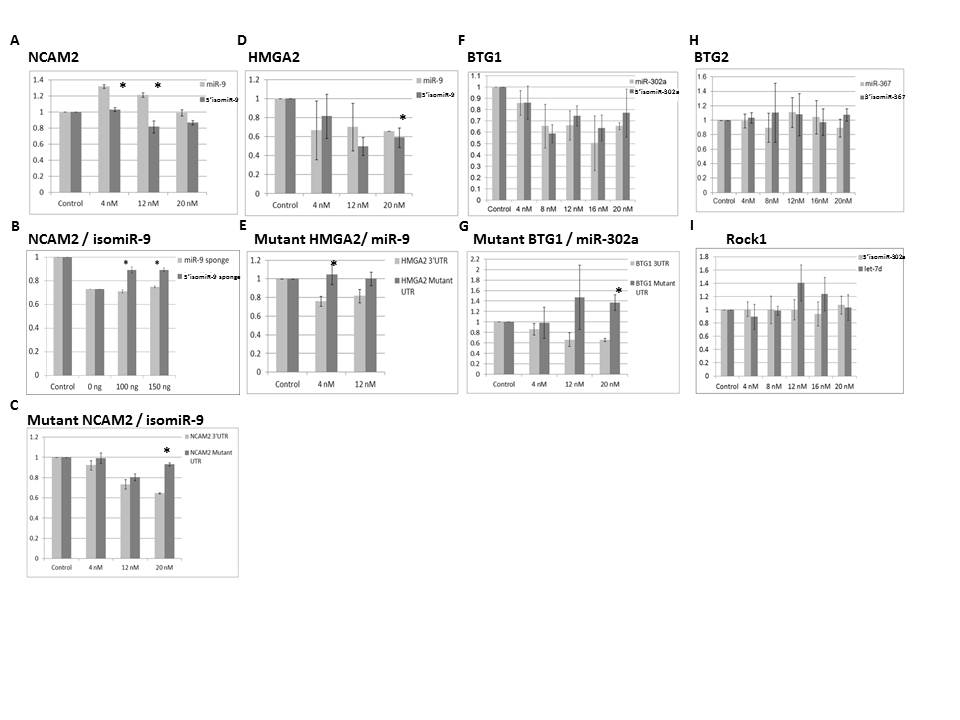

Supplement: SUPPLEMENTARY DATA [file supp_gku656_nar-02457-a-2013-File018.jpg]
