# Supplementary material for: 5′ isomiR variation is of functional and evolutionary importance
Source: Nucleic Acids Res. 2014 Jul 23;42(14):9424–35. doi: 10.1093/nar/gku656 (PMC4132760; doi:10.1093/nar/gku656)
Supplement: SUPPLEMENTARY DATA [file supp_gku656_nar-02457-a-2013-File019.docx]

**A (mir-2).**

bmo-mir-2a-1 Reads bmo-mir-2a-2 Reads

UAUCACAGCCA.... 123 UAUCACAGCCA.... 270

UCACAGCCA.... 600 UCACAGCCA.... 76

**B (mir-10).**

bfl-mir-10a-5p Reads cqu-mir-10-5p Reads

GUACCCUGUAG.... 346 UACCCUGUAGA.... 5

UACCCUGUAG.... 50 ACCCUGUAGA.... 33

mmu-mir-10a-5p Reads dme-mir-10a-5p Reads

AUACCCUGUAG.... 628

UACCCUGUAG.... 213140 UACCCUGUAGA.... 52

ACCCUGUAG.... 12036 ACCCUGUAGA.... 9254

**C (mir-22, 745, 980).**

cte-mir-745a Reads cte-mir-745b Reads

AGCUGCCUGGU.... 2 GAGCUGCCUGGU... 11

Bfl-mir-22-3p Reads dme-mir-980-3p

AAGCUGCCAGA.... 932 UAGCUGCCUUGU.... 61170

AGCUGCCAGA.... 43 AGCUGCCUUGU.... 308

**D (mir-133).**

dme-mir-133-3p mmu-mir-133b-3p

UUUGGUCCCCU.... 7 UUUGGUCCCCU.... 91811

UUGGUCCCCU.... 40030 UUGGUCCCCU.... 5641

**E (mir-137).**

dme-mir-137-3p mmu-mir-137

UUAUUGCUUGA.... 7 UUAUUGCUUAA.... 59525

UAUUGCUUGA.... 1577 UAUUGCUUAA.... 7850

**F (mir-210)**

mmu-mir-210-3p Reads cqu-mir-210-3p Reads

ACUGUGCGUGU.... 5835 CUUGUGCGUGU.... 637

CUGUGCGUGU.... 68651 CUGUGCGUGU.... 388

ame-mir-210-3p

CUUGUGCGUGU.... 1715

UUGUGCGUGU.... 2097
